# Supplementary material for: Severe Impairment of Left Ventricular Regional Strain in STEMI Patients Is Associated with Post-Infarct Remodeling
Source: J Clin Med. 2022 Sep 12;11(18):5348. doi: 10.3390/jcm11185348 (PMC9505824; doi:10.3390/jcm11185348)
Supplement: Supplementary file 1 [file jcm-11-05348-s001.zip › jcm-1854636-supplementary.pdf]

Supplementary Table S1. Therapy at discharge and after one year in patients with and without left ventricular remodeling.

	**Overall patients**	**LVR patients**	**Non-LVR patients**	**P value**
Therapy at discharge				
Beta-blockers (n)	156 (88.1%)	27 (90%)	122 (87.1%)	0.66
Statins (n)	175 (98.9%)	30 (100%)	138 (98.6%)	0.51
ACEIs/ARBs (n)	153 (86.4%)	24 (80%)	126 (90%)	0.12
MRAs (n)	20 (11.3%)	6 (20%)	10 (7.1%)	0.029
Warfarin (n)	11 (6.3%)	3 (10%)	8 (5.8%)	0.39
NOACs (n)	0	0	0	-
Cardioaspirin (n)	173 (100%)	30 (100%)	136 (100%)	-
Clopidogrel (n)	27 (15.3%)	5 (16.7%)	20 (14.3%)	0.73
Ticagrelor (n)	110 (62.1%)	19 (63.3%)	88 (62.9%)	0.96
Prasugrel (n)	38 (21.5%)	6 (20%)	31 (22.1%)	0.79
PPIs (n)	120 (67.8%)	21 (70 %)	92 (65.7%)	0.65
Therapy at one year				
Beta-blockers (n)	135 (82.8%)	24 (88.9%)	111 (81.6%)	0.36
Statins (n)	156 (95.7%)	27 (100%)	129 (94.9%)	0.22
ACEIs/ARBs (n)	149 (91.4%)	25 (92.6%)	124 (91.2%)	0.81
MRAs (n)	20 (12.3%)	8 (29.6%)	12 (8.8%)	0.003
Warfarin (n)	9 (5.6%)	1 (3.7%)	8 (5.9%)	0.64
NOACs (n)	2 (1.2%)	0	2 (1.5%)	0.52
Cardioaspirin (n)	144 (88.3%)	24 (88.9%)	120 (88.2%)	0.92
Clopidogrel (n)	18 (11%)	5 (18.5%)	13 (9.6%)	0.17
Ticagrelor (n)	16 (9.8%)	3 (11.1%)	13 (9.6%)	0.81
Prasugrel (n)	7 (4.3%)	5 (3.7%)	2 (7.4%)	0.38
PPIs (n)	86 (57.0%)	14 (58.3%)	72 (56.7%)	0.88

ACEI, angiotensin converting enzyme inhibitor; ARB, angiotensin II receptor blocker; LVR, left ventricular remodeling; MRA, mineralocorticoid receptor antagonist; NOAC, new oral anticoagulant; PPI, proton pump inhibitor.

Supplementary table S2. Univariate logistic regression analysis for left ventricular remodeling.

	N	OR	95% CI	P value
Infarct site	176	0.57	0.35–0.91	0.019
Final TIMI flow	177	0.61	0.14–2.6	0.506
Time from FMC to PCI (minutes)	177	1	1–1	0.041
Time from symptoms' onset to diagnosis ≥ 3h	177	2.818	1.08–7.38	0.035
Staged PCI	177	0.624	0.27–1.5	0.277
Residual vessels after staged PCI	177	1.2	0.72–2	0.473
Complete revascularization	177	0.77	0.09–6,64	0.812
Number of deseased coronary vessels	177	1.14	0.7–1.87	0.583
ECG ST-resolution >50%	177	0.32	0.14–0.71	0.005
BSA (m^2)	177	0.66	0.11–3.87	0.643
BMI (Kg/m^2)	177	1	0.92–1.1	0.915
Age (years)	177	1	0.97–1	0.862
Sex (male)	177	0.58	0.25–1.32	0.195
Systolic blood pressure (mmHg)	130	0.99	0.97–1	0.162
Heart rate (bpm)	104	0.99	0.96–1.03	0.802
Killip class 3–4	177	5.07	0.97–26.49	0.054
Culprit coronary vessel	176	0.73	0.48–1.13	0.158
First MI (no recurrence)	177	2.35	0.87–6.37	0.09
Hypertension	177	0.43	0.17–1.1	0.069
Dyslipidemia	177	1.54	0.68–3.47	0.298
Diabetes	177	0.828	0.31–2.24	0.71
Smoke	177	0.6	0.27–1.33	0.208
CV Family history	177	0.64	0.28–1.5	0.298
ACEIs/ARBs	177	2.25	0.79–6.44	0.131
MRAs	177	3.25	1.08–9.78	0.036
GFR (mL/min)	177	1	0.99–1	0.738
Total colesterol (mg/dl)	172	1	0.99–1	0.858
LDL (mg/dl)	163	1	0.99–1	0.99
Peak CK-MB (ng/mL)	177	1	1–1.01	0.002
Peak TnI (ng/mL)	176	1.04	1.02–1.06	<0.0005
Glicate Hb (mmol/mol)	164	1.03	1–1.1	0.02
ESV (mL) (for 5 mL increase)	177	1.15	1.05–1.27	0.004
EF (%)	177	0.88	0.83–0.92	<0.0005
GLS (%)	177	1.49	1.26–1.76	<0.0005
%SAS (for 5% increase)	173	1.29	1.16–1.42	<0.0005
MR grade	162	1.4	0.8–2.45	0.238
Left atrial volume index (mL/m^2)	164	1	0.96–1	0.673
WMSI	176	1.29	1.14–1.45	<0.0005
%WMA	176	1.07	1.04–1.1	<0.0005
E/A ratio	151	1.58	0.74–3.4	0.234
E/e' ratio	153	0.96	0.83–1.12	0.631

ACEI, angiotensin converting enzyme inhibitor; ARB, angiotensin II receptor blocker; BMI, body mass index; bpm, beat per minute; BSA, body surface area; CCA, circumflex coronary artery; CK-MB, creatinekinase-MB; CI, confidence interval; COPD, chronic obstructive pulmonary disease; CV, cardiovascular; FMC, first medical contact; ECG, electrocardiogram; EDV, end-diastolic volume; EF, ejection fraction; ESV, end-systolic volume; GFR, glomerular filtration rate; GLS, global longitudinal strain; Hb, hemoglobin; IA, intermediate coronary artery; LAD, left anterior descending artery; LDL, low density lipoprotein; LVR, left ventricular remodeling; MI, myocardial infarction; MR, mitral regurgitation; MRA, mineralocorticoid receptor antagonist; PCI, percutaneous coronary intervention; RCA, right coronary artery; %SAS, percentage of severely altered strain; TIMI, thrombolysis in myocardial infarction; Tn, troponin; %WMA, percentage of the extent of wall motion abnormalities; WMSI, wall motion score index.

Supplementary table S3. Baseline characteristics of patients with and without composite clinical events (death for all causes, re-hospitalization for acute coronary syndrome or heart failure).

	**Patients with clinical events** (n 41)	**Patients without clinical events** (n 136)	**P value**
Age (years)	72 (62–78)	62 (54–70)	<0.0005
Males (n)	28 (68.3%)	100 (73.5%)	0.511
BSA (m^2)	2 (1.8–2.1)	2 (1.8–2.1)	0.588
BMI (Kg/m^2)	26.7 (24.2–33)	26.4 (24.7–29.7)	0.574
Smoke (n)	20 (48.8%)	81 (59.6%)	0.222
Diabetes (n)	18 (43.9%)	18 (13.2%)	<0.0005
CV Family history (n)	40 (29.4%)	10 (24.4%)	0.531
Hypertension (n)	32 (78%)	79 (58.1%)	0.021
Dyslipidemia (n)	20 (48.8%)	60 (44.1%)	0.599
COPD (n)	4 (9.8%)	1 (0.7%)	0.002
LDL (mg/dl)	112.5 (83–139.9)	127.4 (109.4–161)	0.017
Glicate Hb (mmol/mol)	43 (39–51)	39 (36–42)	0.001
GFR (mL/min)	77.9 (44.1–89)	92.5 (67.3–111.5)	0.002
Peak CK-MB (ng/mL)	145.2 (69–287.6)	159.6 (93,4–249,8)	0.886
Peak TnI (ng/mL)	39.4 (15.8–73.9)	39.1 (18.9–69.7)	0.909
Systolic blood pressure (mmHg)	130 (119–140)	130 (120–145)	0.736
Heart rate (bpm)	80 (74–89)	70 (61–80)	0.022
Killip class			
1–2	37 (90.2%)	131 (96.3%)	0.120
3–4	4 (9.8%)	5 (3.7%)	
Previous infarct	12 (29.3%)	13 (9.6%)	0.001
Infarct site			
-Anterior (n)	21 (52.5%)	58 (42.6%)	0.116
-Lateral (n)	11 (27.5%)	25 (18.4%)	
-Inferior (n)	8 (20%)	46 (33.8%)	
-Inferior and right ventricle (n)	0	7 (5.1%)	
Culprit coronary vessel			
-LAD (n)	20 (50%)	57 (41.9%)	0.07
-CCA (n)	7 (17.5%)	25 (18.4%)	
-RCA (n)	10 (25%)	51 (37.5%)	
-Left main (n)	1 (2.5%)	3 (2.2%)	
-IA (n)	2 (5%)	0	
Number of deseased coronary vessels			
-One (n)	13 (31.7%)	52 (38.2%)	0.449
-Two (n)	13 (31.7%)	48 (35.3%)	
-Three (n)	15 (36.6%)	36 (26.5%)	
Final TIMI flow			
-0 (n)	1 (2.4%)	0	0.214
-1 (n)	0	1 (0.7%)	
-2 (n)	2 (4.9%)	3 (2.2%)	
-3 (n)	38 (92.7%)	132 (97.1%)	
Time from FMC to PCI (minutes)	58 (46–92)	56 (42–90)	
Time from symptoms' onset to diagnosis			
< 3h	31 (75.6%)	119 (87.5%)	0.063
≥ 3h	10 (24.4%)	17 (12.5%)	
ECG ST-resolution (>50%) (n)	22 (53.7%)	88 (64.7%)	0.201
Primary PCI (n)	41 (100%)	136 (100%)	-
Staged PCI (n)	12 (29.3%)	55 (40.4%)	0.196
Complete revascularization	2 (4.9%)	5 (3.7%)	0.729
Residual vessels after staged PCI	1 (1–2)	1 (1–2)	-

BMI, body mass index; bpm, beat per minute; BSA, body surface area; CCA, circumflex coronary artery; CK-MB, creatinekinase-MB; COPD, chronic obstructive pulmonary disease; CV, cardiovascular; FMC, first medical contact; ECG, electrocardiogram; GFR, glomerular filtration rate; Hb, hemoglobin; IA, intermediate coronary artery; LAD, left anterior descending artery; LDL, low density lipoprotein; PCI, percutaneous coronary intervention; RCA, right coronary artery; TIMI, Thrombolysis In Myocardial Infarction; Tn, troponin. For each variable, the median value with interquartile range is reported, unless differently indicated.

Supplementary table S4. Echocardiographic characteristics of patients with and without clinical events (death for all causes, re-hospitalization for acute coronary syndrome or heart failure).

	**Patients with clinical events**	**Patients without clinical events**	**P value**
EDV (mL)	97 (86–118)	99 (86–116)	0.914
ESV (mL)	53 (43–70)	50 (42–63)	0.232
EF (%)	41.9 (33.8–50)	49.5 (43.1–53.9)	0.001
GLS (%)	−11.2 (−14.6–−9)	−13.6 (−16.2–−11.1)	0.001
%SAS	41 (23–65)	23 (6–53)	0.004
Left atrial volume index (mL/m^2)	31 (26–37)	29 (24–33)	0.047
MR grade			
-No MR (n)	10 (25.6%)	47 (38.2%)	0.009
-Mild (n)	16 (41%)	63 (51.2%)	
-Moderate (n)	12 (30.8%)	12 (9.8%)	
-Severe (n)	1 (2.6%)	1 (0.8%)	
WMSI	1.875 (1.5–2.125)	1.625 (1.375–1.937)	0.033
% WMA	37.5 (18.75–50)	31.25 (12.5–37.5)	0.046
E/A ratio	0.8 (0.6–1.1)	0.8 (0.7–1.1)	0.355
E/e' ratio	12.8 (10–15.1)	8.8 (6.8–10.3)	0.015

EDV, end-diastolic volume; EF, ejection fraction; ESV, end-systolic volume; GLS, global longitudinal strain; MR, mitral regurgitation; %SAS, percentage of severely altered strain; %WMA, percentage of the extent of wall motion abnormalities; WMSI, wall motion score index. For each variable, the median value with interquartile range is reported, unless differently indicated.

Supplementary table S5. Univariate baseline variables cox analysis for composite clinical events (death for all causes, re-hospitalization for acute coronary syndrome or heart failure).

	N	HR	95% CI	P value
Infarct site	176	0.69	0.48–0.99	0.045
Final TIMI flow	177	0.49	0.25–0.96	0.037
Time from FMC to PCI (minutes)	177	1	1–1	0.679
Time from symptoms' onset to diagnosis ≥ 3h	177	0.48	0.24–0.99	0.046
Staged PCI	177	0.60	0.3–1.21	0.153
Residual vessels after staged PCI	177	1.61	1.09–2.36	0.016
Complete revascularization	177	1.63	0.39–6.76	0.502
Number of deseased coronary vessels	177	1.27	0.87–1.87	0.212
ECG ST-resolution >50%	177	0.6	0.32–1.13	0.208
BSA (m^2)	177	0.37	0.09–1.45	0.153
BMI (Kg/m^2)	177	1.02	0.96–1.09	0.518
Age (years)	177	1.06	1.03–1.09	<0.001
Sex (male)	177	1.30	0.67–2.51	0.436
Systolic blood pressure (mmHg)	130	1.00	0.99–1.02	0.962
Heart rate (bpm)	104	1.02	1–1.05	0.039
Killip class (3–4)	177	2.85	1.01–8.01	0.048
Culprit coronary vessel	176	0.94	0.68–1.3	0.7
First MI (no recurrence)	177	0.27	0.14–0.53	<0.001
COPD	177	6.46	2.27–18.4	<0.001
Diabetes	177	4.32	2.30–8.13	<0.001
Smoke	177	0.72	0.39–1.33	0.300
Hypertension	177	2.23	1.07–4.68	0.033
Dylipidemia	177	0.88	0.48–1.63	0.697
CV Family history	177	0.90	0.45–1.79	0.757
ACEIs/ARBs	177	0.17	0.09–0.32	<0.0005
MRAs	177	3.79	1.89–7.59	<0.0005
GFR (mL/min)	177	0.98	0.98–0.99	0.003
Total colesterol (mg/dl)	172	0.99	0.98–1	0.007
LDL (mg/dl)	163	0.99	0.98–1	0.009
Peak CK-MB (ng/mL)	177	1.00	1–1	0.962
Peak TnI (ng/ml)	176	1.00	0.99–1.01	0.597
Glicate Hb (mmol/mol)	164	1.02	1.01–1.04	0.007
ESV (mL)	177	1.02	1.01–1.03	0.005
EF (%)	177	0.92	0.88–0.95	<0.001
GLS (%)	177	1.21	1.10–1.34	<0.001
%SAS	173	1.02	1.01–1.03	<0.001
MR grade	162	1.77	1.17–2.69	0.007
Left atrial volume index (mL/m^2)	164	1.03	1.00–1.06	0.026
WMSI	176	4.33	1.83–10.2	<0.001
%WMA	176	1.03	1.01–1.05	0.003
E/A ratio	151	0.56	0.21–1.47	0.239
E/e' ratio	70	1.06	0.99–1.13	0.088
LVR	170	2.53	1.19–5.34	0.015

ACEI, angiotensin converting enzyme inhibitor; ARB, angiotensin II receptor blocker; BMI, body mass index; bpm, beat per minute; BSA, body surface area; CCA, circumflex coronary artery; CK-MB, creatinekinase-MB; CI, confidence interval; COPD, chronic obstructive pulmonary disease; CV, cardiovascular; FMC, first medical contact; ECG, electrocardiogram; EDV, end-diastolic volume; EF, ejection fraction; ESV, end-systolic volume; GFR, glomerular filtration rate; GLS, global longitudinal strain; Hb, hemoglobin; IA, intermediate coronary artery; LAD, left anterior descending artery; LDL, low density lipoprotein; LVR, left ventricular remodeling; MI, myocardial infarction; MR, mitral regurgitation; MRA, mineralocorticoid receptor antagonist; PCI, percutaneous coronary intervention; RCA, right coronary artery; %SAS, percentage of severely altered strain; TIMI, Thrombolysis In Myocardial Infarction; Tn, troponin; %WMA, percentage of the extent of wall motion abnormalities; WMSI, wall motion score index.
